# Identification of Candidate Genes Downstream of TLR4 Signaling after Ozone Exposure in Mice: A Role for Heat-Shock Protein 70

**DOI:** 10.1289/ehp.1003326

**Published:** 2011-05-04

**Authors:** Alison K. Bauer, Elizabeth A. Rondini, Kristin A. Hummel, Laura M. Degraff, Christopher Walker, Anne E. Jedlicka, Steven R. Kleeberger

**Affiliations:** 1 Department of Pathobiology and Diagnostic Investigation, Michigan State University, East Lansing, Michigan, USA; 2 Laboratory of Respiratory Biology, National Institute of Environmental Health Sciences, National Institutes of Health, Department of Health and Human Services, Research Triangle Park, North Carolina, USA; 3 Department of Molecular Microbiology and Immunology, Johns Hopkins University, Baltimore, Maryland, USA

**Keywords:** heat-shock protein 70, inflammation, ozone, pulmonary, toll-like receptor 4, transcriptomics

## Abstract

Background: Toll-like receptor 4 (TLR4) is involved in ozone (O_3_)-induced pulmonary hyperpermeability and inflammation, although the downstream signaling events are unknown.

Objectives: The aims of our study were to determine the mechanism through which TLR4 modulates O_3_-induced pulmonary responses and to use transcriptomics to determine potential TLR4 effector molecules.

Methods: C3H/HeJ (HeJ; *Tlr4* mutant) and C3H/HeOuJ (OuJ; *Tlr4* normal) mice were exposed continuously to 0.3 ppm O_3_ or filtered air for 6, 24, 48, or 72 hr. We assessed inflammation using bronchoalveolar lavage and molecular analysis by mRNA microarray, quantitative RT-PCR (real-time polymerase chain reaction), immunoblots, immunostaining, and ELISAs (enzyme-linked immunosorbent assays). B6-*Hspa1a/Hspa1btm1Dix/*NIEHS (*Hsp70–/–*) and C57BL/6 (B6; Hsp70+/+ wild-type control) mice were used for candidate gene validation studies.

Results: O_3_-induced TLR4 signaling occurred through myeloid differentiation protein 88 (MyD88)-dependent and -independent pathways in OuJ mice and involved multiple downstream pathways. Genomewide transcript analyses of lungs from air- and O_3_-exposed HeJ and OuJ mice identified a cluster of genes that were significantly up-regulated in O_3_-exposed OuJ mice compared with O_3_-exposed HeJ mice or air-exposed controls of both strains; this cluster included genes for heat-shock proteins (e.g., *Hspa1b*, *Hsp70*). Moreover, O_3_-induced inflammation, MyD88  up-regulation, extracellular-signal–related kinase-1/2 (ERK1/2) and activator protein-1 (AP-1) activation, and kerotinocyte-derived chemokine (KC) protein content were significantly reduced in *Hspa1a/Hspa1b^tm1Dix^* (*Hsp70*^–/–^) compared with *Hsp70*^+/+^ mice (*p* < 0.05).

Conclusions: These studies suggest that HSP70 is an effector molecule downstream of TLR4 and is involved in the regulation of O_3_-induced lung inflammation by triggering similar pathways to TLR4. These novel findings may have therapeutic and preventive implications for inflammatory diseases resulting from environmental exposures.

Ozone (O_3_) is a principal oxidant of pollution and is generated when nitrous oxides and volatile organic compounds react with singlet and molecular oxygen in the presence of ultraviolet light (Mudway and Kelly 2000). In the United States, an estimated one-half of individuals exposed to O_3_ are at risk of developing pulmonary disease (American Lung Association 2011), and approximately 118 million U.S. residents live in cities out of attainment (i.e., in which the O_3_ levels are higher than federal regulations) or in regions that approach or exceed the National Ambient Air Quality Standard (NAAQS) set for O_3_ [U.S. Environmental Protection Agency (EPA) 2011]. O_3_ exposure may lead to premature death (Bell et al. 2005; Jerrett et al. 2009), dyspnea (Horstman et al. 1990), wheezing and coughing (Triche et al. 2006), increased susceptibility to lung infection (Hollingsworth et al. 2007), increased risk of asthma attacks (Burnett et al. 1997; Desqueyroux et al. 2002; Gent et al. 2003), reduced birth weight (Salam et al. 2005), and new-onset asthma in children living in regions with high concentrations of O_3_ (McConnell et al. 2002). In 2008, the NAAQS was reduced from 0.080 ppm to 0.075 ppm because of increased concern about human health risks, and further reduction of O_3_ levels in the U.S. is currently under review (U.S. EPA 2010).

In mice, O_3_ causes pulmonary inflammation [indicated by polymorphonuclear leukocyte (PMN) infiltration] and hyperpermeability [indicated by increased proteins in bronchoalveolar lavage fluid (BALF)] (Kleeberger et al. 1993a, 1993b, 1997). A genomewide linkage analysis found a susceptibility quantitative trait locus for O_3_-induced hyperpermeability (Kleeberger et al. 2000), and toll-like receptor 4 (*Tlr4*) was identified as a candidate gene. *Tlr4* has been implicated in innate immunity and endotoxin [specifically lipopolysaccharide (LPS)] susceptibility (Kopp and Medzhitov 1999; Poltorak et al. 1998; Qureshi et al. 1999). Significantly higher BALF protein concentrations and lung tissue *Tlr4* mRNA were found in C3H/HeOuJ (OuJ; *Tlr4* normal) mice after exposure to O_3_, compared with C3H/HeJ (HeJ; *Tlr4* dominant negative mutant) mice (Kleeberger et al. 2000). In addition, *Tlr4* deficiency protected against O_3_-induced airway hyperresponsiveness (Hollingsworth et al. 2004). Together, these results indicate that the chromosome 4 quantitative trait locus explains a substantial portion of the genetic variance in O_3_-induced hyperpermeability and support *Tlr4* as a susceptibility gene.

TLR4 protein is activated by the binding of ligand(s), which leads to the recruitment of adaptor molecules, including myeloid differentiation protein 88 (MyD88) (Akira and Takeda 2004). For example, LPS in a complex with LPS-binding protein, CD14, and myeloid differentiation protein-2 binds to TLR4 and is therefore an exogenous ligand (Poltorak et al. 1998). The MyD88-dependent pathway then signals through mitogen-activated protein kinase (MAPK), nuclear factor κB (NFκB), and/or activator protein-1 (AP-1) pathways to induce downstream genes such as *Tnfα* (tumor necrosis factor α), *Il1β* (interleukin-1β), and *Kc* (keratinocyte-derived chemokine) in response to ligands such as LPS (Akira and Takeda 2004). A MyD88-independent pathway signals through TRAM [Toll-interleukin-1 receptor domain containing adaptor protein-inducing interferon-β (TRIF)-related adaptor molecule] and TRIF binding, which mediates the activation of interferon regulatory factor 3, leading to the induction of interferon-α,β–inducible genes, such as *Ip10* (IFNγ-inducible 10 kDa protein) (Akira and Takeda 2004).

The objective of this study was to identify the pathways through which TLR4 mediates O_3_-induced lung inflammation and injury and to validate the functional role of downstream effectors. We used a transcriptomics approach to address the hypothesis that TLR4-specific changes in gene expression associate with differential susceptibility to O_3_-induced pulmonary responses in OuJ and HeJ mice. We then tested one of these pathways [heat-shock protein 70 (HSP70)] using a knockout mouse model to confirm the functional importance of HSP70 to O_3_ responsiveness.

## Materials and Methods

*Animals and O_3_ exposures.* C3H/HeJ (HeJ) and C3H/HeOuJ (OuJ) mice (males 6 weeks of age) were purchased from Jackson Laboratories (Bar Harbor, ME). B6;129S7-*Hspa1a/Hspa1b^tm1Dix^*/Mmcd on a B6129SvF1 background, as described previously (Hunt et al. 2004), were purchased from the Mutant Mouse Regional Resource Center (University of North Carolina–Chapel Hill, Chapel Hill, NC). These B6;129S7-*Hspa1a/Hspa1b^tm1Dix^*/Mmcd mice were crossed onto the C57BL/6J (B6) background (Jackson Laboratories) until the sixth generation (N6) [called B6-*Hspa1a/Hspa1b^tm1Dix^*/NIEHS (*Hsp70*^–/–^)], when they were intercrossed with siblings. These mice are thus 96.9% B6 (Silver 1995). Male age-matched (6–8 weeks of age) wild-type controls (C57BL/6J; *Hsp70*^+/+^; Jackson Laboratories) were also used for these studies. Mice were fed laboratory chow (NIH-07; Zeigler Brothers, Gardners, PA) and given water *ad libitum* before and during exposures. OuJ and HeJ mice were exposed to O_3_ as described previously (Yoon et al. 2007), and all other exposure studies were done at the Michigan State University Containment Facility as described previously (Wagner et al. 2003). Mice were exposed continuously in whole-body chambers to filtered air or 0.3 ppm O_3_ for 6, 24, 48, or 72 hr (23.5 hr/day). Immediately after exposure, mice were euthanized by sodium pentobarbital (104 mg/kg). Time points used for each phenotype were based on kinetics identified in previous studies [chemokines (Yoon et al. 2007) and transcription factors, gene expression, and MAPK (Cho et al. 2007)]. All animals were treated humanely and with regard for alleviation of suffering. All animal use and procedures were approved by the institutional animal care and use committees of the National Institute of Environmental Health Sciences and Michigan State University.

*BALF analysis.* For some OuJ and HeJ mice and all *Hsp70*^+/+^ and *Hsp*^–/–^ mice, the left lobe of the lung was clamped and the right lung lobes were lavaged based on body weight (17.5 mL/kg). We performed lavage analysis to determine cell differentials and total protein (indicator of lung hyperpermeability) as described elsewhere (Bauer et al. 2010; Kleeberger et al. 1997, 2000). The left lobes (all strains) were either snap frozen in liquid nitrogen or inflation fixed in 10% neutral buffered formalin and processed for histology.

*RNA extraction and Affymetrix GeneChip array processing.* Total RNA was extracted from left lobes of lung from OuJ and HeJ mice exposed to air or 6, 24, or 48 hr O_3_ (*n* = 3/treatment group) and homogenized in TRIZOL reagent (Invitrogen, Gaithersberg, MD) [for details, see Supplemental Material, p. 3 (doi:10.1289/ehp.1003326)]. Right lung lobes from the same animals were used for quantitative real-time polymerase chain reaction (qRTPCR) confirmation. Processing of templates for GeneChip Analysis followed methods described in the GeneChip Expression Analysis Technical Manual, Revision Three (Affymetrix Inc. 2005–2009).

*Transcriptomic analysis.* CEL format files were imported into GeneSpring (version 7.0; Silicon Genetics, Redwood City, CA) for statistical analyses and characterization of data [for details, see Supplemental Material pp. 3–5 (doi:10.1289/ehp.1003326)]. All samples were normalized in GeneSpring to OuJ (wild-type) air controls (Cho et al. 2005), and *k*-means cluster analyses were performed.

*qRTPCR confirmation of array data and TLR4 downstream adaptor molecules.* qRTPCR  was performed using either the Taqman assay or the Sybr green assay (both from Applied Biosystems, Foster City, CA) following the manufacturer’s instructions [for details of qRTPCR, see Supplemental Material, p. 5; for primers, see Supplemental Material, Table 2 (doi:10.1289/ehp.1003326)].

*Immunohistochemical detection of HSP70.* HSP70 was detected in lung sections from O_3_-exposed OuJ and HeJ mice using a specific HSP70 antibody (EMD Chemicals, Gibbstown, NJ) and a labeled streptavidin-biotin (LSAB) secondary antibody (DAKO, Carpinteria, CA). Immunodetection of HSP70 was evaluated as previously described (Cho and Kleeberger 2007; Cho et al. 2005).

*NF*κ*B and phosphorylated c-Jun (p-c-Jun) nuclear binding activity.* We used 8 μg nuclear protein prepared from left lung lobes of mice 6 and 24 hr after O_3_ exposure (Active Motif, Carlsbad, CA) to determine specific binding of p-c-Jun and NFκB p65 proteins using transcription factor ELISA (enzyme-linked immunosorbent assay; TransAM kit, Active Motif), similar to that described for previous studies (Rondini et al. 2010).

*Immunoblot detection of MAPK and HSP70.* For immunoblot detection of MAPK and HSP70, we used primary antibodies specific for MAPK (Cell Signaling, Danvers, MA), β-actin (Sigma, St. Louis, MO), and HSP70 (EMD Chemicals), followed by horseradish peroxidase secondary antibodies (Pierce; Thermo Fisher, Rockford, IL). Following protein extraction and separation, we used 75–100 μg protein from control and O_3_ exposed lungs for immunoblotting according to previously published methods (Bauer et al. 2005; Cho et al. 2005; Rondini et al. 2010). Densitometry was then performed using the BioRad ChemiDoc illumination system with Quality One software (Bio-Rad, Carlsbad, CA).

*ELISA for kerotinocyte-derived chemokine (KC) and macrophage inflammatory protein-2 (MIP-2).* We analyzed KC (CXCL1) and MIP-2 in BALF using ELISA kits from R&D Systems (Minneapolis, MN) according to manufacturer’s instructions.

*Statistics.* Data are expressed as mean ± SE. We used two-way analysis of variance to evaluate the effects of exposure and strain on BALF phenotypes, qRTPCR, ELISAs, transcription factors, and immunoblotting studies. Student Newman-Keuls test was used for *a posteriori* comparisons of means; statistical significance was defined as *p* < 0.05. All analyses were performed using a commercial statistical analysis package (SigmaStat, version 3.5; Jandel Scientific Software, San Rafael, CA).

## Results

**Figure 1:**
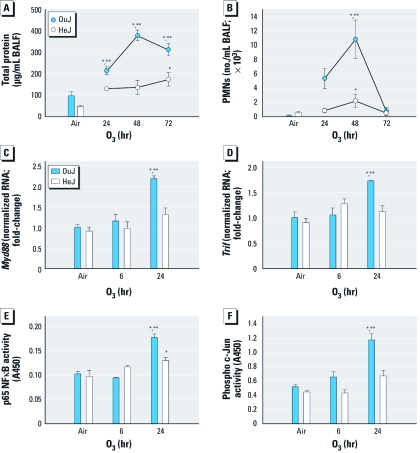
Differential response (mean ± SE) to air or 0.3 ppm O_3_ in lungs of OuJ and HeJ mice. Total protein concentration (a marker of lung permeability; *A*) and number of PMNs recovered (*B*) from the BALF (*n* = 3–7 mice/group, repeated once). Expression of *Myd88* (*C*) and *Trif* mRNA (*D*) (normalized RNA; *n* = 3–5 mice/group, repeated once). Differential NFκB (*E*) and AP-1 (*F*) DNA binding activity (measured at a wavelength of 450 nm) (*n* = 3–4 mice/group, repeated once). *p < 0.05 compared with air-exposed controls. **p < 0.05 compared with O_3_-exposed HeJ mice.

*TLR4 signaling in response to O_3_.* We found significantly greater mean total protein (at 24, 48, and 72 hr) and numbers of BALF PMNs (at 48 hr) in O_3_-exposed OuJ mice compared with OuJ controls and both air- and O_3_-exposed HeJ mice (Figure 1A,B), as described previously (Kleeberger et al. 2000). Epithelial cell numbers in BALF were also significantly different (24 and 72 hr) between OuJ and HeJ strains [see Supplemental Material, Table 3 (doi:10.1289/ehp.1003326)]. After 24 hr O_3_ exposure, transcript levels of the adaptor molecules *Myd88* and *Trif* were up-regulated in OuJ mice compared with the three other treatment groups (OuJ controls and both air- and O_3_-exposed HeJ mice; Figure 1C,D), similar to changes found previously for *Tlr4* mRNA expression (Kleeberger et al. 2000). Transcription factors NFκB (p65 subunit) and AP-1 (p-c-Jun) were likewise significantly higher after 24 hr O_3_ exposure in OuJ mice compared with O_3_-exposed HeJ mice or air-exposed controls of both strains(Figure 1E,F; *p* < 0.05).

**Figure 2:**
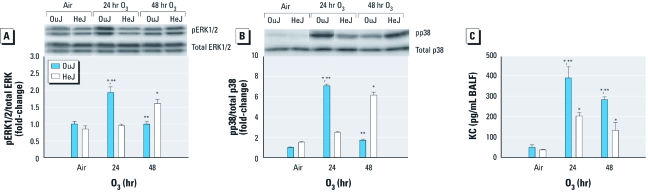
MAPKs and chemokine pathways downstream of TLR4 in OuJ and HeJ mice in response to air or 0.3 ppm O_3_. Phosphorylated MAPK activity [pERK1/2 (*A*) and pp38 (*B*)] detected by immunoblotting; proteins were normalized to the unphosphorylated form of the same protein (total MAPK) and are expressed as fold change (mean ± SE) relative to air control OuJ mice (*n* = 3 mice/group; repeated three times; duplicates were run on the same gel). (*C*) KC protein levels (mean ± SE) in BALF detected by ELISA (*n* = 3–6 mice/group, repeated once). *p < 0.05, compared with air-exposed controls. **p < 0.05, compared with O_3_-exposed HeJ mice.

Three primary MAPKs—ERK1/2 (extracellular-signal–related kinase-1/2), JNK (c-Jun N-terminal kinase), and p38—are involved in response to O_3_ (Cho et al. 2007) and LPS-mediated TLR4 signaling in the lung (Chanteux et al. 2007; Fang et al. 2007). After 24 hr O_3_ exposure, ERK1/2 and p38 proteins were significantly elevated in OuJ mice compared with OuJ controls and both air- and O_3_-exposed HeJ mice, but after 48 hr of exposure ERK1/2 and p38 were significantly increased in O_3_-exposed HeJ mice compared with O_3_-exposed OuJ mice or controls of both strains (Figure 2A,B). JNK activity was unchanged in both strains after O_3_ exposure (data not shown). Furthermore, neutrophil chemoattractant KC (CXCL1) protein levels were significantly elevated in OuJ compared with HeJ mice after 24 and 48 hr O_3_ exposure (Figure 2C); *Kc* transcript levels were also elevated after 24 hr O_3_ (data not shown). We found no effects of O_3_ on protein levels of MIP-2, another neutrophil chemoattractant, in either strain (data not shown).

*Transcriptomic analysis to identify TLR4 effector genes. k*-Means clustering identified five clusters after the initial filtering, which determined 200 transcripts with significant interactions for strain and time with 2-fold changes in gene expression (*p* < 0.05; data not shown). We focused subsequent analyses on 39 genes that were distributed in three distinct cluster patterns [clusters 2, 4, and 5; see Supplemental Material, Excel Table 1A–C (doi:10.1289/ehp.1003326)]. In cluster 2 (24 genes; see Supplemental Material, Table 4A and Supplemental Material, Figure 1A), expression of transcripts was significantly greater in OuJ mice after 24 and 48 hr O_3_ exposure compared with air-exposed controls of both strains and O_3_-exposed HeJ mice. Analysis using the Database for Annotation, Visualization, and Integrated Discovery [DAVID (Huang et al. 2009a, 2009b); see Supplemental Material, Excel Table 1C] identified protein folding, response to heat and stress, response to temperature stimulus, chaperone, and response to protein stimulus (*p*-values ranged from 4.35 × 10^–9^ to 4.0 × 10^–5^) as major functional categories. Five heat-shock proteins in the antigen processing and presentation KEGG pathway [*Hspa1b*, *Hsp90aa1*, *Hsp90ab1*, *Hspa5*, *Hspa8*U; (Kyoto Encyclopedia of Genes and Genomes; Kanehisa Laboratories 2010)] were particularly notable (see Supplemental Material, Figure 1B).

In HeJ mice, cluster 4 transcripts [10 genes; see Supplemental Material, Table 4B (doi:10.1289/ehp.1003326)] were expressed at significantly higher levels in mice exposed to O_3_ for 24 hr compared with air-exposed controls, and most genes were decreased after 48 hr. In contrast, we found minimal changes in OuJ mice (data not shown). DAVID analysis categorized 6 of the 10 genes in this cluster (e.g., *Cdkn1*, *Mt2*, *Mt1*) into metal-binding, transition-metal ion-binding, zinc ion-binding, and cation-binding functions (*p*-values ranged from < 0.007 to < 0.044; for complete analysis, see Supplemental Material, Excel Table 1D). Cluster 5 (5 genes; see Supplemental Material, Table 4C) contained transcripts that were significantly greater in HeJ than in OuJ mice after 24 and 48 hr O_3_ exposure. Four of five of these genes are related to inflammation and immune response. DAVID analysis for cluster 5 (see Supplemental Material, Excel Table 1E) identified one functional category (secretion; *p* < 0.03) for three of these genes (*Saa3*, *Cxcl5*, and *Timp1*).

**Figure 3:**
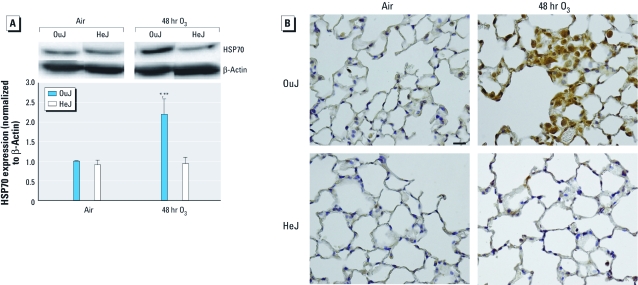
Immunodetection of 70 HSP70 protein in lungs of OuJ and HeJ mice in response to 48 hr exposure to air or 0.3 ppm O_3_. (*A*) HSP70 expression detected by immunoblotting; HSP70 protein was normalized to β-actin and is expressed as fold change relatative to the OuJ air controls (mean ± SE; *n* = 3 mice/group; repeated once). (*B*) Photomicrographs showing immunohistochemical staining of HSP70. Bar = 20 µM. *p < 0.05, compared with air-exposed controls. **p < 0.05, compared with O_3_-exposed HeJ mice.

*Candidate gene validation.* To confirm some of the genes identified using expression profiling, we performed qRTPCR on genes from all three focus clusters in the same samples used for the microarray analysis [Supplemental Material, Figure 2 (doi:10.1289/ehp.1003326)]. *Hspa1b* mRNA expression did not significantly change after 48 or 72 hr O_3_ exposure in HeJ mice, in contrast to the array results (see Supplemental Material, Figure 2B). Because a significant number of genes were identified in the heat-shock protein functional category, we focused on HSP70 (the protein encoded by *Hspa1b*) for further validation. In HeJ mice, HSP70 protein expression was not changed after O_3_ exposure. However, HSP70 protein expression was significantly elevated in O_3_-exposed OuJ mice compared with OuJ controls and both air- and O_3_-exposed HeJ mice (Figure 3A). HSP70 immunostaining confirmed up-regulation and localization in alveolar macrophages and epithelial cells in OuJ mice (Figure 3B).

**Figure 4:**
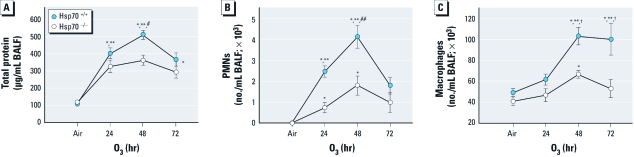
Inflammatory parameters measured in BALF from *Hsp70*^–/–^ and *Hsp*^+/+^ mice after exposure to air or 0.3 ppm O_3_. (*A*) Total protein concentration. (*B*) Total number of PMNs. (*C*) Total number of macrophages in BAL from *Hsp70*^–/–^ and *Hsp70*^+/+^ mice. Values shown are mean ± SE; *n* = 3–6 mice/group, repeated four times. *p < 0.05, compared with air-exposed controls. **p < 0.05, compared with O_3_-exposed *Hsp70*^–/–^ mice. ^#^*p* < 0.05, compared with 72 hr O_3_ exposure. ^##^*p* < 0.05, compared with 24 or 72 hr O_3_ exposure. ^†^*p* < 0.05, compared with 24 hr O_3_ exposure.

*HSP70 involvement in O_3_-induced responses.* To further investigate the role of HSP70 in this model, we exposed *Hsp70*^–/–^ and *Hsp70*^+/+^ mice to air and 0.3 ppm O_3_. Relative to *Hsp70*^+/+^ mice, BALF total protein (at 24 and 48 hr), PMNs (at 24 and 48 hr), and macrophages (at 48 and 72 hr) were significantly reduced in *Hsp70*^–/–^ mice after O_3_ exposure (Figure 4). Epithelial cell numbers were not significantly different between strains [see Supplemental Material, Table 3 (doi:10.1289/ehp.1003326)]. Histopathology also demonstrated increased cellularity and thickening of the airways in *Hsp70*^+/+^ mice (data not shown). We found significantly increased *Hspa1b* gene expression in *Hsp70*^+/+^ mice after 48 hr of O_3_ compared with *Hsp70*^+/+^ controls (data not shown).

**Figure 5:**
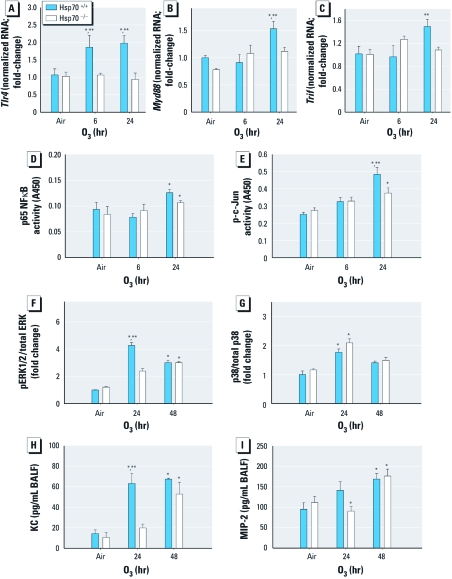
TLR4 and adaptor mRNA expression and pathways downstream of TLR4, including transcription factors, MAPKs, and chemokines, in control and O_3_-exposed *Hsp70*^+/+^ and *Hsp70*^–/–^ mice. Expression of *Tlr4 *(*A*), *Myd88 *(*B*), and *Trif *(*C*) transcripts (*n* = 3–5 mice per treatment group; repeated twice). Nuclear binding activity of p65 NFκB (*D*) and p‑c‑Jun (*E*) (*n* = 3–4 mice per treatment group; repeated once). Quantitation of phosphorylated MAPK activity [pERK1/2 (*F*) and pp38 (*G*)]. Proteins were normalized to the unphosphorylated form of the same protein (total MAPK); *n* = 3 mice/group, repeated three times (duplicate samples per study were run on the same gel followed by normalization to total MAPK). Protein levels of KC (*H*) and MIP‑2 (*I*) in BALF (*n* = 3–4 mice/group; repeated once). Values shown are mean ± SE. *p < 0.05, compared with air-exposed controls. vs O_3_; **p < 0.05, compared with O_3_-exposed *Hsp70*^–/–^ mice.

After 24 hr exposure, transcript levels  of *Myd88* were significantly increased in O_3_-exposed *Hsp70*^+/+^ mice compared with O_3_-exposed *Hsp70*^–/–^ mice and controls of both strains (Figure 5B). *Trif* mRNA expression was significantly higher in *Hsp70*^+/+^ mice than in *Hsp70*^–/–^ mice (24 hr O_3_ exposure), but it was not significantly different from controls of either strain (Figure 5D). Importantly, *Tlr4* was also significantly increased in the *Hsp70*^+/+^ but not *Hsp70*^–/–^ mice after 6 and 24 hr O_3_ exposure (Figure 5A). NFκB p65 binding activity was significantly increased in both genotypes after 24 hr exposure to O_3_ compared with controls, but we found no significant differences between genotypes (Figure 5D). Binding activity of p-c-Jun was significantly increased in O_3_-exposed animals of both genotypes compared with controls, but was significantly higher in *Hsp70*^+/+^ mice than in *Hsp70*^–/–^ mice (Figure 5E). ERK1/2 protein was also significantly elevated in O_3_-exposed *Hsp70*^+/+^ mice compared with *Hsp70*^+/+^ controls and both air- and O_3_-exposed *Hsp70*^–/–^ mice (Figure 5F), whereas p38 was elevated in both strains after 24 hr exposure to O_3_ compared with controls (Figure 5G). JNK was unchanged in both strains (data not shown), similar to OuJ and HeJ mice. In the 24-hr exposure group, KC protein levels were significantly elevated in O_3_-exposed *Hsp70*^+/+^ mice compared with O_3_-exposed *Hsp70*^–/–^ mice and air controls of both genotypes (Figure 5H; *p* < 0.05). In the 48-hr exposure group, KC levels in O_3_-exposed mice of both genotypes were significantly greater than those in respective air controls. We found a significant decrease in MIP-2 protein levels in O_3_-exposed *Hsp70*^–/–^ mice compared with the corresponding controls in the 24-hr group, but MIP-2 levels were significantly increased in both genotypes after 48 hr of O_3_ exposure compared with air controls of both genotypes (Figure 5I).

## Discussion

The objective of the present study was to determine the mechanisms through which TLR4 modulates O_3_-induced inflammation and injury. TLR4 signaling in response to O_3_ involved significant elevations in MyD88-dependent and -independent (TRIF) pathways in *Tlr4* (OuJ) but not *Tlr4* mutant (HeJ) mice, suggesting that both effector pathways are involved in mediating O_3_-induced inflammation and hyperpermeability. Additionally, we observed significant strain differences in NFκB and AP-1 transcription factors, MAPK [phosphorylated ERK (pERK) and phosphorylated p38 (pp38)], and the chemokine KC, further supporting a role for TLR4-induced signaling pathways in O_3_-induced pulmonary responses. Using microarrays, we identified heat-shock proteins as one of the primary Gene Ontology (GO) categories (Gene Ontology Consortium 2011) significantly up-regulated in OuJ mice relative to HeJ mice after O_3_ exposure. *Hsp70*^–/–^ mice were used to validate the importance of HSP70 in response to O_3_. Significantly greater inflammation and hyperpermeability in *Hsp70*^+/+^ mice compared with *Hsp70*^–/–^ mice after 48 hr O_3_ exposure were consistent with a role for HSP70 in TLR4-mediated effects in this model. Further, MyD88-dependent signaling that appears to involve ERK1/2, AP-1, and KC was greater in *Hsp70*^+/+^ than in *Hsp70*^–/–^ mice after exposure. Collectively, the timing of up-regulation in OuJ mice (24–48 hr) suggests that HSP70 is induced after the initial TLR4 activation and that HSP70 subsequently contributes to further activation of the TLR4 pathway in an MyD88-dependent manner. Additional temporal similarities between the *Tlr4* mutant and *Hspa1b*-deficient models, such as downstream signaling events (i.e., AP-1, ERK1/2, and KC), also support a link between these two pathways and provide evidence to support HSP70 as a downstream mediator of TLR4 in O_3_-induced pulmonary injury and inflammation. However, it is important to note that although there are many similarities in O_3_ sensitivity between the B6 (*Hspa1b* wild-type) and OuJ (*Tlr4* wild-type) mice, genes other than *Tlr4* likely contribute to the enhanced O_3_ sensitivity in these two strains relative to *Hspa1b-*deficient and *Tlr4* mutant mice.

This is the first study to suggest a panel of HSPs (*Dnaja1*, *Dnajb4*, *Hsp90aa1*, *Hsp90ab1*, *Hspa1b*, *Hspa5*, and *Hspa8*) as effector genes in response to O_3_-induced TLR4 signaling. The HSP70 family of proteins is highly conserved evolutionarily and includes multiple genes (e.g., *Hspa9*, *Hspa5*, *Hspa1a*, *Hspa1b*, *Hspa8*) (Daugaard et al. 2007; Huang et al. 2001). *Hspa1a* and *Hspa1b* are located on mouse chromosome 17 and human chromosome 6, respectively (Daugaard et al. 2007; Huang et al. 2001) and encode nearly identical proteins (Hsp70.1 and Hsp70.3, 99% homologous). *Hspa1b* was also recently identified as a candidate susceptibility gene in the O_3_ susceptibility quantitative trait locus *Inf2* on chromosome 17 (Bauer et al. 2010). Hsp70.1 and Hsp70.3 (HSP70) have been implicated in stress regulation, including heat shock, oxidative stress, and other environmental stresses, such as ultraviolet light exposure (Daugaard et al. 2007; Su and Gordon 1997; Zhou et al. 1998).

HSP70 is ubiquitously expressed in mouse and human lung (Sartori and Scherrer 2003), and is localized intra- and extracellularly, although the mechanism involved in HSP70 secretion is unclear (Mambula et al. 2007; Wheeler and Wong 2007). Augmented HSP70 expression has been reported in the lungs of individuals with asthma, acute lung injury, respiratory syncytial virus infection, and cancer (Sartori and Scherrer 2003). HSP70 protein expression was also elevated in isolated lavage cells (primarily macrophages) and whole-lung guinea pig and rat homogenates (Su and Gordon 1997) after acute O_3_ exposure (Servais et al. 2005; Su and Gordon 1997). Interestingly, acute O_3_ exposure did not trigger the induction of the Hsp70-1 promoter in a transgenic mouse model (Wirth et al. 2002); however, higher O_3_ levels and different mouse strains were used than in the studies noted above. Rats exposed chronically to O_3_ also had increased HSP70 (Wong et al. 1996); however, primates exposed chronically to O_3_ had repressed HSP70, among other HSPs (Wu et al. 1999). The discrepancy between acute and chronic O_3_ exposure models may reflect differences in species, strain, and/or exposure protocol.

O_3_-induced oxidative stress likely results from cyclical and self-generating reactions forming highly unstable radical and nonradical reactive oxygen species, such as aldehydes, hydroxyl radical, and hydrogen peroxide (Pryor et al. 1995). However, the role of HSP70 in O_3_-induced oxidative stress responses is unclear. Pulmonary HSP70 is induced by cadmium in mice (Wirth et al. 2002), sodium arsenite in guinea pigs (Su and Gordon 1997), and hyperthermia in rats (Wheeler and Wong 2007), among other oxidative stress-inducers, and is hypothesized to elicit a protective or adaptive response in the respiratory epithelium after initial exposure (Wheeler and Wong 2007). The present study has implicated HSP70 as important to the progression of O_3_-induced lung injury and underscores the complex roles of HSPs in mediating oxidant-induced lung injury.

We also found that, in mice with impaired TLR4 signaling (HeJ), the genes involved in mediating the downstream effects in response to O_3_ were primarily in the metal-binding functional category (*Cdkn1*, *Mt1*, *Mt2*). *Mt1*- and *Mt2*-deficient mice are more susceptible to O_3_-induced responses; thus, metallothionein 1 and 2 appear to be protective against the effects of O_3_ (Inoue et al. 2008). Another gene that we previously identified as differentially expressed between OuJ and HeJ mice (*Marco*) is also protective against O_3_-induced lung injury (Dahl et al. 2007).

*In vivo* administration of HSP70 induced the TLR4 pathway (Chase et al. 2007); when applied to human bone-marrow–derived PMNs *in vitro*, HSP70 induced the production of KC in a TLR4-dependent manner (Wheeler et al. 2009). In our model, we hypothesize that HSP70 is in part responsible for triggering the initial increase in BALF inflammatory phenotypes observed in the OuJ and *Hsp70*^+/+^ mice. We provide evidence that after the initial activation, HSP70 signals through MyD88 only; thus, another pathway is likely involved in TLR4 signaling involving the MyD88-independent (TRIF) pathway. We observed a delayed ERK1/2 activation response in both models (*Tlr4-* and *Hsp70*-deficient), another indication that the two models are linked temporally. p38 was differentially expressed between OuJ and HeJ mice but not between *Hsp70*^+/+^ and *Hsp70*^–/–^ mice, suggesting that p38 is induced through the MyD88-independent pathway. However, unlike the involvement of JNK downstream of TNF receptor signaling (Cho et al. 2007), JNK does not appear to be involved in the TLR4 pathway. Additionally, it appears that MIP-2 induction is independent of TLR4 and HSP70 in this model.

## Conclusion

The present study demonstrates a novel finding for TLR4 effector genes and suggests that these pathways, including HSPs (e.g., *Hspa1b*, *Hsp90ab1*) and metal binding (e.g., *Mt1*, *Mt2*), should be investigated with regard to their roles in determining susceptibility to O_3_-induced lung inflammation and injury in humans. We also provide *in vivo* evidence that HSP70 can trigger activation of multiple signaling pathways known to be downstream of TLR4, as well as other TLRs, although the precise mechanism of the interaction between them remains unclear. Recent evidence on the adverse human health effects of pollution, including both O_3_ and particulate matter, supports the need for additional studies to identify individuals at risk.

## Supplemental Material

(68 KB) PDF

(236 KB) XLS
