# Supplementary material for: Identification of Candidate Genes Downstream of TLR4 Signaling after Ozone Exposure in Mice: A Role for Heat-Shock Protein 70
Source: Environ Health Perspect. 2011 May 4;119(8):1091–7. doi: 10.1289/ehp.1003326 (PMC3237361; doi:10.1289/ehp.1003326)
Supplement: (68 KB) PDF [file ehp.1003326.s001.pdf]

Supplemental Material

Identification of Candidate Genes Downstream of TLR4 Signaling after Ozone Exposure in Mice: A Role for Heat Shock Protein 70

Alison K. Bauer[1], Elizabeth A. Rondini[1], Kristin A. Hummel[1], Laura M. Degraff[2], Christopher Walker[2], Anne E. Jedlicka[3], Steven R. Kleeberger[2]

[1]Department of Pathobiology and Diagnostic Investigation, Michigan State University, East Lansing, MI 48824; [2]Laboratory of Respiratory Biology, National Institute of Environmental Health Sciences, National Institutes of Health, Research Triangle Park, NC 27709, [3]Department of Molecular Microbiology and Immunology, Johns Hopkins University, Baltimore, MD 21205

Correspondence should be sent to current address:
Alison K. Bauer, Ph.D.
Department of Environmental and Occupational Health
Mail Stop B119-V20, Rm. V20-3125
Pharmacy and Pharmaceutical Sciences Building
University of Colorado Anschutz Medical Campus
12850 East Montview Boulevard
Aurora, CO 80045
Phone number: (303) 724-6297
FAX number: (303) 724-4495
e-mail address: alison.bauer@ucdenver.edu

Table of Contents

Supplemental Material, Methods *pages 3-5*

Supplemental Material, Table 1 Legend *page 6*
(Supplemental Table 1 is a separate Excel file)

Supplemental Material, Table 2 *page 7*

Supplemental Material, Table 3 *page 8*

Supplemental Material, Table 4 *pages 9-13*

Supplemental Material, Figure 1 *pages 14*

Supplemental Material, Figure 2 *page 15*

Supplemental Material, Methods:

RNA extraction and Affymetrix GeneChip array processing. Briefly, lung tissue was homogenized in 2ml Trizol with a Tekmar Tissumizer and saw-tooth generator. One ml of homogenate was subsequently processed according to the manufacturer's (Invitrogen) protocol with the following minor modifications. Two microliters of 5mg/ml glycogen was used as a carrier for the isopropanol precipitation, duration of the isopropanol precipitation was increased to overnight, and all centrifugation times were increased to 15 minutes. RNA pellets were resuspended in Nuclease-free water. Quantitation was performed using a Beckman DU680 or NanoDrop spectrophotometer, and quality assessment was determined by RNA Nano LabChip analysis on an Agilent Bioanalyzer 2100. A Qiagen RNeasy total RNA cleanup protocol was subsequently performed followed by re-quantitation by spectophotometry. For each treatment group, subsequent processing and GeneChip analysis was performed in triplicate.

Affymetrix GeneChip array analysis: Double stranded cDNA was synthesized from 7.5 micrograms of total RNA and was purified by Phenol/Chloroform Extraction followed by Ethanol precipitation. Phase Lock Gels (Eppendorf) were used in conjunction with the extraction protocol and glycogen was utilized during the precipitation. Precipitated and washed cDNAs were resuspended in RNase-free water. cRNA was synthesized from one half of the double-stranded cDNA by *in vitro* transcription (IVT) using the BioArray High Yield RNA Transcript Labeling Kit (Enzo Life Sciences), according to the manufacturer's recommended protocol. Resultant cRNAs were purified by column purification with the GeneChip Sample Cleanup Module (Affymetrix), and quantified. 15 micrograms of cRNA were fragmented by metal-induced hydrolysis in fragmentation buffer (250mM Tris acetate pH 8.1, 150 mM MgOAc, 500mM KOAc) at 94° C for 35 minutes. Aliquots of pre- and post-fragmentation cRNAs were reserved for quality assessment by RNA Nano LabChip analysis on an Agilent Bioanalyzer 2100. Hybridization cocktails were prepared as recommended for arrays of

“Standard” format including incubation at 94°C for 5 minutes and 45°C for 5 minutes, and centrifugation at maximum speed for 5 minutes prior to pipetting into the GeneChips (Affymetrix Murine Genome MOE430A). Hybridization was performed at 45°C for 16 hours at 60 rpm in the Affymetrix rotisserie hybridization oven. The signal amplification protocol for washing and staining of eukaryotic targets was performed in an automated fluidics station (Affymetrix FS450) as described in the Affymetrix Technical Manual, Revision Three.

The arrays were then transferred to the GCS3000 laser scanner (Affymetrix) and scanned at an emission wavelength of 570nm at 2.5 μm resolution. Intensity of hybridization for each probe pair was computed by GCOS 1.1 software. Primary analysis consisted of a quality assessment of the hybridization of each sample. Ratios of signal for probe sets at 5’ and 3’ regions of housekeeping genes were calculated and monitored as an indication of transcript quality for each sample. The hybridization intensity for each probe array was used in an expression algorithm which designates each transcript as Present (P), Absent (A) or Marginal (M).

Transcriptomic analysis. Stringent filtering of genes was done using GeneSpring 7.0 (Silicon Genetics, Redwood City, CA), including elimination of absent flags in all groups and inclusion of only those genes with significant interactions for strain and time ($p<0.05$, 2-way ANOVA; Benjamin and Hochberg False Discovery Rate test for the multiple comparisons). We then identified genes with 2-fold changes in expression in OuJ versus HeJ mice at each time point (Supplemental Materials, Excel Table 1, Tab A, 200 genes). *k*-means clustering was performed on these 200 transcripts to identify groups with similar expression patterns (5 clusters for the 200 genes, data not shown).

Transcriptomic pathway and functional analyses. We focused on the three most interesting clusters (2, 4, and 5; see Supplemental Material, Excel Table 1, Tab C, E, and G) for further analysis. The genes from all three clusters were then combined and subjected to analysis using

functional classification available at DAVID (Database for Annotation, Visualization and Integrated Discovery http://david.abcc.ncifcrf.gov/) (see Supplemental Material, Excel Table 1, Tab B). Each individual cluster was also analyzed individually using DAVID (Supplemental Material, Table 1, Tab D, F, and H). Genes in pathways with enrichment *p*-values below $p < 0.05$ were selected. All genes in clusters 2, 4, and 5 are shown in Supplemental Material, Table 2 and organized by the top functional categories per cluster. The raw microarray data have been deposited into NCBI Gene Expression Omnibus (GEO, http://www.ncbi.nlm.nih.gov/geo/, Series GSE20715).

Quantitative real time PCR (qRTPCR) confirmation of array data and TLR4 downstream adaptor molecules. qRTPCR was performed using either Taqman or Sybr green assays on an Applied Biosystems 7900 Prism Sequence Detection System following manufacturer's instructions (probe primer sets are in Supplemental Material, Table 2A and the Sybr green primers in Supplemental Material, Table 2B). Universal master mixes for Taqman and Sybr green were used according to the manufacturer's instructions (User bulletin no. 2, Applied Biosystems Prism 7700 Sequence Detection System). 18S was used to normalize all genes of interest since it did not vary across genotypes or treatments.

Supplemental Material, Table 1 Legend: Comprehensive list of all the microarray data. A. List of 200 transcripts identified using *k*-means clustering that had significant interactions for strain and time ($p < 0.05$), Tab A. Affymetrix ID, gene name, Genbank number, description, gene ontology (GO) biological processes, GO molecular functions, and GO cellular components are listed in order (columns A-G). B-E. DAVID analysis with Category, term, count, percent, p value, genes involved in that category, list, population hits, population total, fold enrichment, Bonferroni, Benjamini, and FDR are listed in this order (columns A-M). B. Comprehensive DAVID analysis for all 200 transcripts categorized by functional group, Tab B. C. Cluster 2 DAVID analysis categorized by functional group, Tab C. D. Cluster 4 DAVID analysis categorized by functional group Tab D. E. Cluster 5 DAVID analysis categorized by functional group, Tab E. Please see file named Supplemental Material, Excel Table 1.

Supplemental Material, Table 2. Primers used for quantitative RT-PCR.

A: Primer probe identification for qRTPCR analysis.

**Name**	**Probe ID***	**Accession no.**
Eln	Mm00514670_m1	NM_007925.3
Hspa1b	Mm03038954_s1	NM_010478.2
Hspcb	Mm00833431_g1	NM_008302.3
Mt1	Mm00496660.g1	NM_013602.3
Saa3	Mm00441203_m1	NM_011315.3
Sprr1a	Mm00442225_m1	NM_009264.2
18S	Hs99999901_s1	X03205.1

* Probes sets from purchased from Applied Biosystems.

B: Primers used for Sybr green qRTPCR analysis.

**Name**	**Direction**	**Sequence**	**Accession no.**
MyD88	forward	CCG GCA ACT AGA ACA GAC	BC005591.1
	reverse	TCT GTT GGA CAC CTG GAG ACA	
Tlr4	forward	ATT GAA GAC AAG GCA ATG GCA T	AF095353
	**reverse**	GTG AGC CAC ATT GAG TTT CTT	
Trif	forward	GAC ACA CCA GAC TGG AGT TG	BC094338.1
	reverse	GAG GCC AGC TTA TCT CCT CAG	
18S	forward	GAGAAACGGCTACCACATCCAA	NR_003278
	reverse	CCTCCAATGGATCCTCGTTAAAG	

Supplemental Material, Table 3. Epithelial cell numbers in the BALF from all four strains of mice exposed to O_3.

		**Ozone exposure (h)**		
**Strain**	Air	24	48	72
OuJ	1.85 ± 1.10	4.25 ± 0.67^{+}	2.39 ± 1.00	7.78 ± 2.24^{*+}
HeJ	0.66 ± 0.26	0.41 ± 0.23	0.51 ± 0.21	0.76 ± 0.42
Hsp70$^{+/+}$	0.73 ± 0.12	2.02 ± 0.79	3.86 ± 1.23	5.24 ± 1.89
Hsp70$^{-/-}$	0.79 ± 0.36	0.93 ± 0.29	2.79 ± 0.61	2.32 ± 1.53

*, $p < 0.05$ for air compared to O_3 exposed + $p < 0.05$ for strain differences between OuJ and HeJ. Cell numbers are cells/ml BALF X 10^3.

Supplemental Material, Table 4: Genes identified using K-means clustering analysis in Genespring that were ≥ 2-fold changes in expression in OuJ and HeJ mice after O_3 exposure*.

A. Cluster 2 genes upregulated to a greater extent in the OuJ mice vs HeJ mice.

Gene symbol	Gene description	Affymetrix probe ID	Major GO[a] categories
Heat shock proteins (alternate names)			
Dnaja1	DnaJ (Hsp40) homolog, subfamily A, member 1	1416288_at	reproduction; protein folding
Dnajb4	DnaJ (Hsp40) homolog, subfamily B, member 4	1431734_a_at	protein folding; metabolic process
Hsp90aa1	heat shock protein 90, alpha (cytosolic), class A member 1	1426645_at; 1437497_a_at	cell activation; immune system process
Hsp90ab1	heat shock protein 90 alpha (cytosolic), class B member 1	1416364_at; 1416365_at	protein folding; nitrogen compound metabolic process
Hspa1b (Hsp70.3)	heat shock protein 1B	1427127_x_at; 1452318_a_at	telomere maintenance; nucleobase, nucleoside, nucleotide and nucleic acid metabolic process
Hspa5 (Grp78)	heat shock 70kD protein 5	1416064_a_at; 1427464_s_at	protein folding; apoptosis
Hspa8 (Hsc70)	heat shock protein 8	1420623_x_at	regulation of progression through cell cycle; protein folding
Ottmusg00000006249	similar to heat shock protein 1, alpha	1438902_a_at	
Enzymes			
Amy-2	amylase 2, pancreatic	1416055_at	carbohydrate metabolic process; metabolic process
Cyp7b1	cytochrome P450, family 7, subfamily b, polypeptide 1	1421074_at	alcohol metabolic process; organic acid metabolic process
Fkbp4	FK506 binding protein 4	1416362_a_at	reproduction; reproductive developmental process
Mat1a	methionine adenosyltransferase I,	1423147_at	one-carbon compound metabolic process; metabolic

	alpha		process
P4ha1	procollagen-proline, 2-oxoglutarate 4-dioxygenase (proline 4-hydroxylase), alpha 1 polypeptide	1426519_at	protein modification process; amino acid and derivative metabolic process
Ptgs2	prostaglandin-endoperoxide synthase 2	1417262_at	prostaglandin biosynthetic process; hair follicle development
Ptp4a1	protein tyrosine phosphatase 4a1	1419024_at	protein modification process; protein amino acid dephosphorylation
Wfdc12	WAP four-disulfide core domain 12	1449191_at	defense response; response to biotic stimulus
Replication and transcription			
Cdc46	minichromosome maintenance deficient 5, cell division cycle 46	1415945_at	nucleobase, nucleoside, nucleotide and nucleic acid metabolic process; DNA metabolic process
Tsc22d2	TSC22 domain family 3	1425281_a_at	nucleobase, nucleoside, nucleotide and nucleic acid metabolic process; transcription
Signal Transduction			
Klra20	killer cell lectin-like receptor subfamily A, member 20	1451942_x_at	
Ms4a62	membrane-spanning 4-domains, subfamily A, member 6D	1419598_at	cell communication; signal transduction
Per2	period homolog 2	1417602_at	two-component signal transduction system; nucleobase, nucleoside, nucleotide and nucleic acid metabolic process
Other			
Angpt1	angiopoietin 1	1421441_at	Angiogenesis; blood vessel development
Ccdc117	cDNA sequence	1452218_at	

2410002O22Rik	BC018601 RIKEN cDNA 2410002O22 gene	1418667_at

*All genes had significant interaction effects for strain and time ($p<0.05$). [a]Based on the top two GO biological functions.

B. Cluster 4 genes up-regulated in HeJ mice more so than in OuJ mice at 24 hr.

Gene symbol	Gene description	Affymetrix probe ID	Major GO[a] categories
Metal Binding			
Admts4	a disintegrin-like and metallopeptidase with thrombospondin type 1 motif, 4	1452595_at	urogenital system development; metanephros development
Cdkn1	cyclin-dependent kinase inhibitor 1A (P21)	1424638_at	
Lox	lysyl oxidase	1416121_at	blood vessel development; vasculature development
Mt1	metallothionein 1	1422557_s_at	cellular ion homeostasis; cellular metal ion homeostasis
Mt2	metallothionein 2	1428942_at	
Peg3	Peg3 mRNA for zinc finger protein	1433924_at	carbohydrate metabolic process; aminoglycan metabolic process
Inflammation and immune response			
Arnt1	aryl hydrocarbon receptor nuclear translocator-like	1425099_a_at	immune system process; immune response
Retnla	resistin like alpha	1449015_at	acute inflammatory response; proteolysis
Other			
Acot1	acyl-CoA thioesterase 1	1422997_s_at	organic acid metabolic process; lipid metabolic process
Eln	elastin	1420854_at	organelle

			organization and biogenesis; cytoskeleton organization and biogenesis

*All genes had significant interaction effects for strain and time (p<0.05). [a] Based on the top two GO biological functions.

C. Cluster 5 genes upregulated in the HeJ at both 24 and 48 hr vs OuJ mice.

**Gene symbol**	**Gene description**	**Affymetrix probe ID**	**Major GO[a] Categories**
Inflammation and immune responses			
Cxcl5	chemokine (C-X-C motif) ligand 5	1419728_at	immune system process; chemotaxis
Marco	macrophage receptor with collagenous structure	1449498_at	transport; ion transport
Saa3	serum amyloid A 3	1450826_a_at	acute inflammatory response; response to stress
Timp1	tissue inhibitor of metalloproteinase 1	1460227_at	immune system process; immune system development
Other			
Sprr1a	small proline-rich protein 1A	1449133_at	multicellular organismal development; ectoderm development

*All genes had significant interaction effects for strain and time (p<0.05). [a] Based on the top two GO biological functions.
